# Cyclone: open-source package for simulation and analysis of finite dynamical systems

**DOI:** 10.1093/bioinformatics/btad634

**Published:** 2023-10-19

**Authors:** Elena S Dimitrova, Adam C Knapp, Brandilyn Stigler, Michael E Stillman

**Affiliations:** Mathematics Department, California Polytechnic State University, San Luis Obispo, CA 93407, United States; Department of MD-Pulmonary Laboratory for Systems Medicine, University of Florida, Gainesville, FL 32610, United States; Department of Mathematics, P.O. Box 750156, Southern Methodist University, Dallas, TX 75275, United States; Department of Mathematics, Cornell University, Ithaca, NY 14853, United States

## Abstract

**Motivation:**

While there are software packages that analyze Boolean, ternary, or other multi-state models, none compute the *complete* state space of function-based models over *any* finite set. Results: We propose Cyclone, a simple light-weight software package which simulates the complete state space for a finite dynamical system over any finite set.

**Availability and implementation:**

Source code is freely available at https://github.com/discretedynamics/cyclone under the Apache-2.0 license.

## 1 Introduction

Finite dynamical systems (FDSs) have become remarkably popular in modeling due to the increasingly important role of interaction-based systems whose behavior can be approximated by a finite number of states. Notable examples include gene regulatory networks (Hinkelmann and Laubenbacher 2013) and discrete event systems (Le Borgne *et al.* 1991).

**Figure 1.**
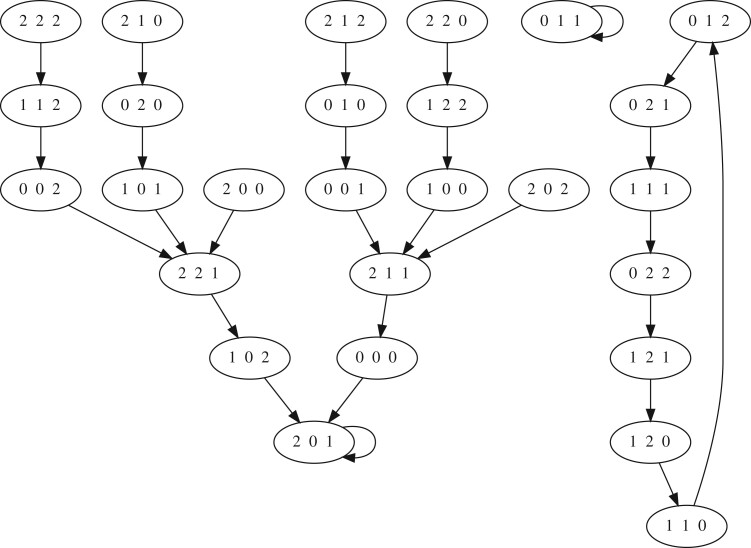
Output state space graph of the input finite dynamical system $F:{\left(Z_{3}\right)}^{3}\to {\left(Z_{3}\right)}^{3}$ where $F=(x_{2}+2,x_{1}+x_{3},x_{1}x_{2}+1)$ and $Z_{3}=\{0,1,2\}$

An FDS in the variables $x_{1},\ldots ,x_{n}$ is a vector function $F=(f_{1},\ldots ,f_{n}):X^{n}\to X^{n}$ and each coordinate function *f_i_* represents how the future value of the *i*-th variable depends on the present values of all variables. For example, a Boolean network is an FDS over X={0,1}. The dynamics of an FDS is generated by the evaluation of F on all *n*-tuples in *X^n^*. The dynamics is represented by the *state space*, a directed graph with vertices in *X^n^* and an edge from *x* to *y* if and only if y=F(x): e.g., see Fig. 1. In an FDS trajectories end in a *limit cycle*, a sequence of states $\left\{s_{1},\ldots ,s_{m}\right\}$ such that $F(s_{i})=s_{i+1}$ for 1≤i<m and $F(s_{m})=s_{1}$. When its size is a power of a prime, *X* can be viewed as a finite field in which functions are represented by polynomials, and algebraic geometry can be used for analysis and model selection (He *et al.* 2019).

There are multiple software platforms available for modeling using FDSs. The Cell Collective (Helikar *et al.* 2012) is an interactive web tool built with a special emphasis on the collaboration of distributed teams to build large Boolean models of molecular networks. The R package BoolNet (Müssel *et al.* 2010) utilizes a convenient approach to build and simulate deterministic Boolean models as well as stochastic ones with randomly varying update schemes for the variables. SteadyCellPhenotype (Knapp *et al.* 2022) analyzes network models under ternary logic. The web tool GINsim (Chaouiya *et al.* 2012) provides a graphical user interface to build and analyze logical models (generalized Boolean networks) via graphs. The web-based application PlantSimLab (Ha *et al.* 2019) uses FDSs as a modeling framework and is targeted at plant biologists for modeling and simulation of molecular networks based on input data formatted as transition tables. Several other recent platforms have been developed; e.g. GeRNet (Dussaut *et al.* 2017), DIN (Forbes *et al.* 2018), *mEPN* scheme (Livigni *et al.* 2018), and MUFINS (Wu *et al.* 2016).

Some of these can deal only with Boolean networks while others can handle multi-state models. All of them have a wide variety of features, whether it is the accommodation of different modeling frameworks, the ability to infer dynamic models from data, or features that allow sharing and distributed model construction. None of them, however, has the capability of taking a system of user-defined functions as input and outputting the complete state space (e.g. neither BoolNet nor GINsim guarantees finding all limit cycles for networks with more than 32 variables (Hinkelmann *et al.* 2011)). Such a capability is of great utility to researchers who build Boolean and multi-state models and wish to have access to the complete state space. This is also useful for educators who teach discrete modeling or incorporate it in student projects.

We note that Cyclone had several web-based precursors over the years. For example, DVD (https://web.archive.org/web/20130602210526/http://dvd.vbi.vt.edu/), Polynome (Dimitrova *et al.* 2011), and ADAM (Hinkelmann *et al.* 2011) were utilized in multiple courses and publications such as Robeva and Hodge (2013). Unfortunately, due to lack of maintenance these tools are now defunct. Cyclone fills in the gap left by them with the same simple-to-use interface but with a dedicated group of developers committed to maintaining functionality. The software is available as an open-source downloadable package via GitHub under the Apache-2.0 license, github.com/discretedynamics/cyclone.

## 2 The package

Cyclone takes as input an FDS defined over the ring $Z_{p}=\{0,\ldots ,p-1\}$ where $Z_{p}$ is the set of integers modulo a positive integer *p* under the ring operations of addition (+) and multiplication (*) modulo *p*. Functions can be written using + and * and can include the list operators MAX and MIN and the generalized logical operator NOT defined by NOT(x)=p−1−x. When *p *=* *2, the Boolean operators AND, OR, and XOR can be used.

Cyclone computes the state space of an FDS using synchronous update scheme. By default it returns two text files: (i) the state space formatted as an input to the dot layout engine in the open source graph visualization software Graphviz (Ellson *et al.* 2002); (ii) a summary of the limit cycles, including the number of components in the state space graph, the size of each component, and the states that form the limit cycle. Cyclone has two options: summary returns only the summary file and trajectory returns only the portion of the state space beginning at a given state and ending at the associated limit cycle.

The software does not impose a limitation on *p* or *n*, nor on the number of variables per function; as such the execution time is determined by the user’s memory and space allocations (as the state space graph has *p^n^* nodes, the corresponding file will have *p^n^* lines). For networks with large state spaces, the above options may be helpful. For example, the following combinations of (*p*, *n*) completed in under 2 min on a Windows 10 laptop with an 11th generation Intel chip running Ubuntu 20.04.3 using the option summary: (2,25), (3,16), and (5,11).

Cyclone is written in C++14 and requires the build system CMake 3.5 or later. The Catch2 framework is used for testing [https://github.com/catchorg/Catch2]. Cyclone has been tested on Debian 11, Ubuntu 20.04, as well as MacOS 11–13.

## 3 Conclusion

Cyclone is a simple light-weight software package that fills in a gap in the available software options for simulating FDSs. It takes as input functions over any finite set and outputs the entire state space or single trajectories. The software is open-source and intended for quick and convenient use in educational and research projects.
